# Identification of Candidate Signature Genes and Key Regulators Associated With Trypanotolerance in the Sheko Breed

**DOI:** 10.3389/fgene.2019.01095

**Published:** 2019-11-14

**Authors:** Yonatan Ayalew Mekonnen, Mehmet Gültas, Kefena Effa, Olivier Hanotte, Armin O. Schmitt

**Affiliations:** ^1^Breeding Informatics Group, Department of Animal Sciences, University of Göttingen, Göttingen, Germany; ^2^Center for Integrated Breeding Research (CiBreed), University of Göttingen, Göttingen, Germany; ^3^Animal Biosciences, National Program Coordinator for African Dairy Genetic Gain, International Livestock Research Institute (ILRI), Addis Ababa, Ethiopia; ^4^Cells, Organisms amd Molecular Genetics, School of Life Sciences, University of Nottingham, Nottingham, United Kingdom; ^5^LiveGene, International Livestock Research Institute (ILRI), Addis Ababa, Ethiopia

**Keywords:** trypanosomiasis, trypanotolerant, selection signature, candidate signature genes, master regulators, overrepresented pathways

## Abstract

African animal trypanosomiasis (AAT) is caused by a protozoan parasite that affects the health of livestock. Livestock production in Ethiopia is severely hampered by AAT and various controlling measures were not successful to eradicate the disease. AAT affects the indigenous breeds in varying degrees. However, the Sheko breed shows better trypanotolerance than other breeds. The tolerance attributes of Sheko are believed to be associated with its taurine genetic background but the genetic controls of these tolerance attributes of Sheko are not well understood. In order to investigate the level of taurine background in the genome, we compare the genome of Sheko with that of 11 other African breeds. We find that Sheko has an admixed genome composed of taurine and indicine ancestries. We apply three methods: (i) The integrated haplotype score (*iHS*), (ii) the standardized log ratio of integrated site specific extended haplotype homozygosity between populations (*Rsb*), and (iii) the composite likelihood ratio (CLR) method to discover selective sweeps in the Sheko genome. We identify 99 genomic regions harboring 364 signature genes in Sheko. Out of the signature genes, 15 genes are selected based on their biological importance described in the literature. We also identify 13 overrepresented pathways and 10 master regulators in Sheko using the TRANSPATH database in the geneXplain platform. Most of the pathways are related with oxidative stress responses indicating a possible selection response against the induction of oxidative stress following trypanosomiasis infection in Sheko. Furthermore, we present for the first time the importance of master regulators involved in trypanotolerance not only for the Sheko breed but also in the context of cattle genomics. Our finding shows that the master regulator Caspase is a key protease which plays a major role for the emergence of adaptive immunity in harmony with the other master regulators. These results suggest that designing and implementing genetic intervention strategies is necessary to improve the performance of susceptible animals. Moreover, the master regulatory analysis suggests potential candidate therapeutic targets for the development of new drugs for trypanosomiasis treatment.

## Introduction

Trypanosomiasis is a disease caused by uni-cellular protozoan parasites which affects the health of humans and livestock. In Africa, this disease is referred to as African animal trypanosomiasis (AAT) (Kristjanson et al., 1999; Shaw et al., 2014). AAT is the major livestock production constraint especially in sub-Saharan African countries. It is mainly caused by *Trypanosoma congolense*, *Trypanosoma vivax*, and *Trypanosoma brucei brucei* (Hoare, 1972; Abebe, 2005, Batista et al., 2011; Yaro et al., 2016). Particularly, *T. congolense* is the most frequent cause of livestock disease in this region (Naessens, 2006). The disease is transmitted from infected animals to healthy animals by tsetse fly as a vector (Welburn et al., 2016). The infected animal shows symptoms such as anemia (Murray et al., 1990; Naessens, 2006), neurological symptoms (Tuntasuvan et al., 1997; Giordani et al., 2016), reduced productivity, infertility, abortion (Barrett and Stanberry, 2009), listlessness, and emaciation (Nantulya, 1986; Batista et al., 2007; Steverding, 2008; Noyes et al., 2011). If not treated, it can lead to death (Kristjanson et al., 1999; Barrett and Stanberry, 2009; Giordani et al., 2016). Hence, this disease has a major economic impact that accounts for an estimated annual loss of US$ 5 billion in sub-Saharan countries (Kristjanson et al., 1999; Giordani et al., 2016).

Ethiopia is located in the eastern part of the tsetse belt. The tsetse fly distribution in the country spans from the south western to the north western regions covering 22,000 km^2^) between longitude 38° and 38° East and latitude 5° and 12° North along river basins (Andrew, 2004; NTTICC, 2004). About 14 million cattle, 7 million horses, 1.8 million camels, and 14 million small ruminants are kept in the infection zone (MoARD, 2004). AAT severely affects the draft power as well as meat and milk production of the animals (Chanie et al., 2013). Therefore, AAT is considered as a major challenge constraining the path toward ensuring food security and combating poverty in this region (Meyer et al., 2018).

Until now, a number of methods have been applied to control the spread of this disease such as trypanocidal drugs, insect traps, and insecticides (Slingenbergh, 1992; Leak et al., 1996; Giordani et al., 2016). But none of these controlling measures has been successful to eradicate the disease. The current situation is deteriorating because of the trypanocidal drug resistance due to inappropriate drug usage. Moreover, pharmaceutical companies are less attracted to invest in new drug discovery and development due to high cost (Codjia et al., 1993; Mulugeta et al., 1997; Kristjanson et al., 1999; Naula and Burchmore, 2003). To control the spread of this disease, Lutje et al. (1996) have suggested a cross breeding strategy between trypanotolerant and trypanosusceptible cattle, together with vector control. Accordingly, Hanotte et al. (2003) performed crossbreeding between the trypanotolerant N’Dama and trypanosusceptible Boran breeds to produce an F_2_ population that shows heterosis. This led to the assumption that an F_2_ cross between trypanotolerant and susceptible breeds could produce a trypanotolerant synthetic breed whose performance would exceed that of either parent. Consequently, marker assisted selection from the F_2_ breed would be the most promising strategy to produce a breed that combines high production and trypanotolerance (Hanotte et al., 2003; Noyes et al., 2011).

In Ethiopia, Sheko shows better trypanotolerance attributes than other breeds such as Abigar and Horro (Lemecha et al., 2006). Sheko is found in the southern region of the Bench Maji Zone, the adjoining areas of Keffa and Shaka and is considered as an endangered breed due to extensive interbreeding with local indicine and sanga breeds (DAGRIS, 2007). Sheko cattle are kept in the tsetse infested regions likely explaining their degree of trypanotolerance (Hanotte et al., 2003; Bahbahani et al., 2018). In order to address the tolerance attributes of the Sheko breed at the molecular level, this study analyzes the genotyping data of the breed to explore the genome for candidate signature genes. The rationale is that natural or artificial selection targets the genome in response to environmental pressures or stresses as shaping adaptation and evolution. This implies that if the new allele of a mutation is beneficial (increases the fitness of their carriers) under certain environmental pressure or stress, then the frequency of these alleles will rapidly increase in the population (Charlesworth, 2007). Under positive selection, strong and long range linkage disequilibrium (LD) and unexpectedly high local haplotype homozygosity might occur in the genome (Gautier and Vitalis, 2012; Bomba et al., 2015).

Likewise, trypanosomiasis is considered as an environmental pressure which plays a major role to create selection signatures in the genome and which is thus leading to breed formation (Kristjanson et al., 1999; Abebe, 2005; Yaro et al., 2016). These signs or traces of selection in the genome could be detected by using a “bottom-up” or a “from genotype to phenotype” approach (McGuire and McGuire, 2008). This study provides traces or signs of positive selection in the genome of Sheko against trypanosomiasis using the “bottom-up” approach. In response to trypanosomiasis as the environmental pressure, the genome of Sheko could undergo changes at the molecular level. With the aim to identify the molecular mechanism of Sheko tolerance, we use extended haplotype homozygosity (EHH; *iHS* and *Rsb*) and spatial distribution of allele frequency [composite likelihood ratio (CLR)] based methods to identify genes that are associated with this selection pressure in the Sheko breed. Combining methods for the detection of selection signature regions has been suggested as a means of increasing the power of the study compared to single analysis (e.g. Ma et al., 2015; Vatsiou et al., 2016).

### Summary of the Analysis Workflow

**Figure 1:**
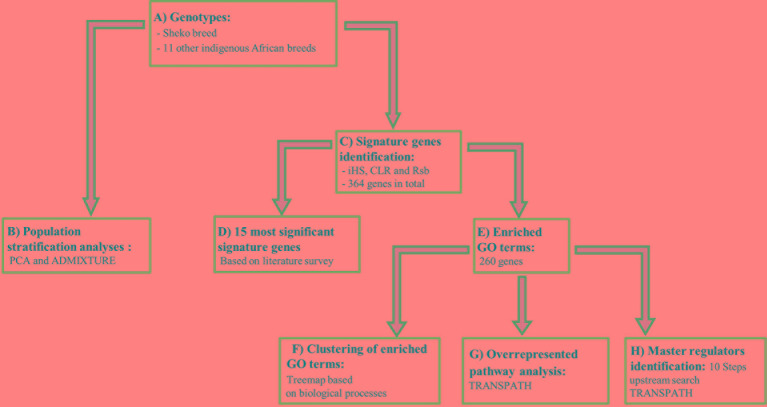
Workflow for the study to identify candidate genes and key regulators that are associated with trypanotolerance in Sheko breed. **(A)** The genotypes of the Sheko and 11 other indigenous African breeds are obtained and quality control filtering is performed. **(B)** The genomic structure of Sheko in comparison to 11 other indigenous African breeds is analyzed using principal component analysis (PCA) and ADMIXTURE. **(C)** The identification of 364 signature genes is performed by *iHS*, CLR, and *Rsb* analyses. **(D)** Among 364 genes, the 15 most significant genes that are associated with trypanotolernace attributes are identified and disclosed. **(E)** Significantly functionally enriched terms [gene ontology (GO) terms] are identified for the 364 signature genes. 260 genes are identified as significantly enriched. **(F)** Using the functionally enriched 260 genes, a treemap is produced based on the biological processes. **(G)** Functionally enriched signature genes (260 genes) are analyzed to identify overrepresented pathways. **(H)** A master regulator network is generated up to 10 steps upstream using functionally enriched signature genes. The treemap, overrepresented pathway, and master regulator analyses were performed in the geneXplain platform.

Our workflow can be divided into two major steps as described below (see also **Figure 1**): 1) We analyzed the genetic relationship and structure of Sheko and 11 other indigenous African breeds using Plink 1.9 and the ADMIXTURE 1.3 software. 2) The identified candidate signature genes were then used in the analysis pipeline comprising the following four sub-steps: i) First, we identified genomic regions and signature genes under positive selection toward trypanotolerance in Sheko using *iHS*, CLR and *Rsb* analyses. As an intermediate result, we present the 15 genes resulting from a literature survey; ii) in the second step, we applied enrichment analysis in gene ontology (GO) terms in the combined gene sets of the three methods and made clusters of enriched GO terms in the form of a treemap using the geneXplain platform; iii) we then identified overrepresented pathways based upon the significant genes found in (ii) using the TRANSPATH database in the geneXplain platform; iv) finally, we identified the master regulators 10 steps upstream in the regulatory hierarchy using the significant genes found in (ii) using the TRANSPATH database in the geneXplain platform.

## Result and Discussion

### The Genetic Relationship and Structure of Cattle Populations

**Figure 2:**
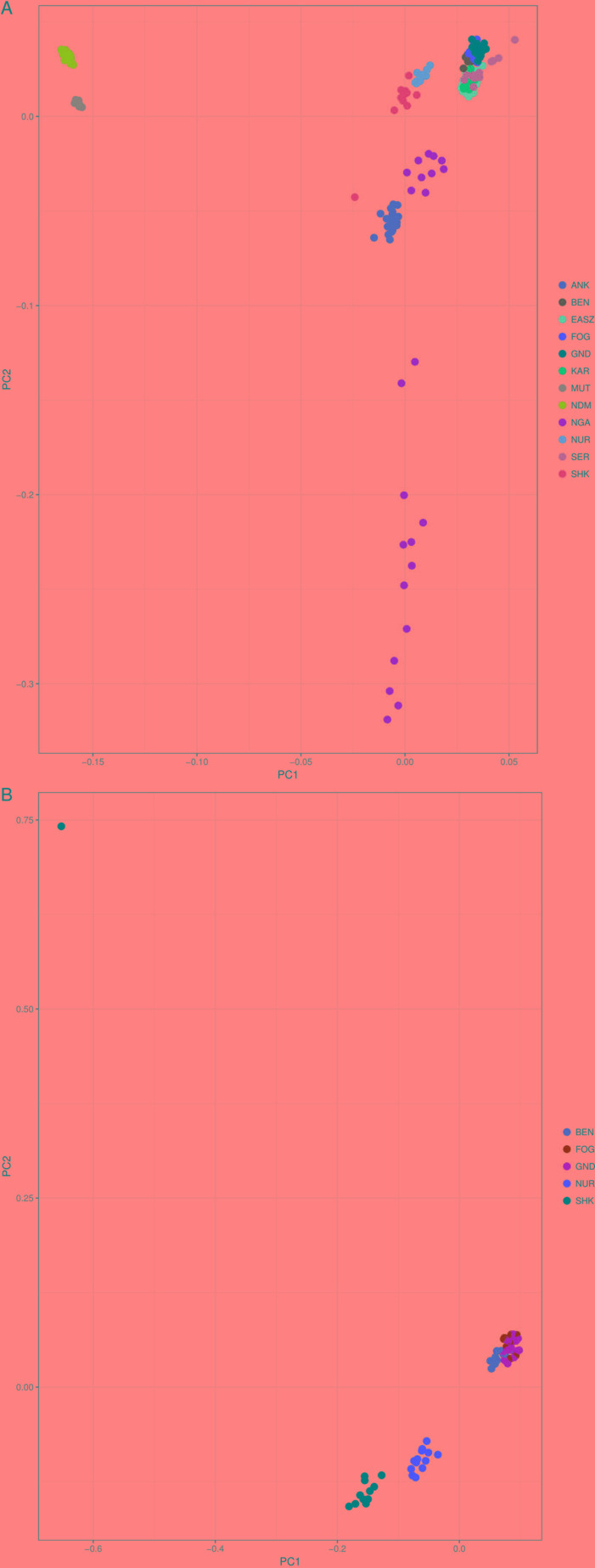
PCA plots of the first two principal components showing the genetic relationship between cattle breeds. **(A)** PCA plot for all cattle breeds included in this study, and **(B)** PCA plot for the Ethiopian cattle breeds. ANK, Ankole; BEN, Benshangul; FOG, Fogera; GND, Gindeberet; KAR, Karamojong; MUT, Muturu; NDM, N’Dama; NGA, Nganda; NUR, Nuer; SER, Serere; SHK, Sheko.

In order to understand the genetic structure of Sheko in comparison with 11 other African breeds, principal component analysis (PCA) was used. The result shows that the first two principal components (PCs), which explain 30.3% and 4.6% of the total variation, distinguishes the African taurine (N’Dama and Muturu) from the African indicine breeds [Benshangul, Serere, Karamojong, East African Shorthorn Zebu (EASZ), Fogera, and Gindeberet] (**Figure 2A**). Moreover, the Sheko, Nganda, Ankole, and Nuer are positioned between the African taurine and the African indicine clusters. These breeds are close to the indicine cluster and thereby support the admixture of more indicine than taurine type genomes in these breeds. The PCA result also shows the highest level of genetic heterogeneity in the Nganda breed which might be caused by ongoing crossbreeding of Nganda with exotic breeds to enhance their productivity (Mwai et al., 2015). We also conducted PCA exclusively for indigenous Ethiopian breeds. The result shows that the Sheko and Nuer form separate groups while the indicine type breeds (Benshangul, Fogera and Gindeberet) form a cluster in both PCs (**Figure 2B**).

**Figure 3:**
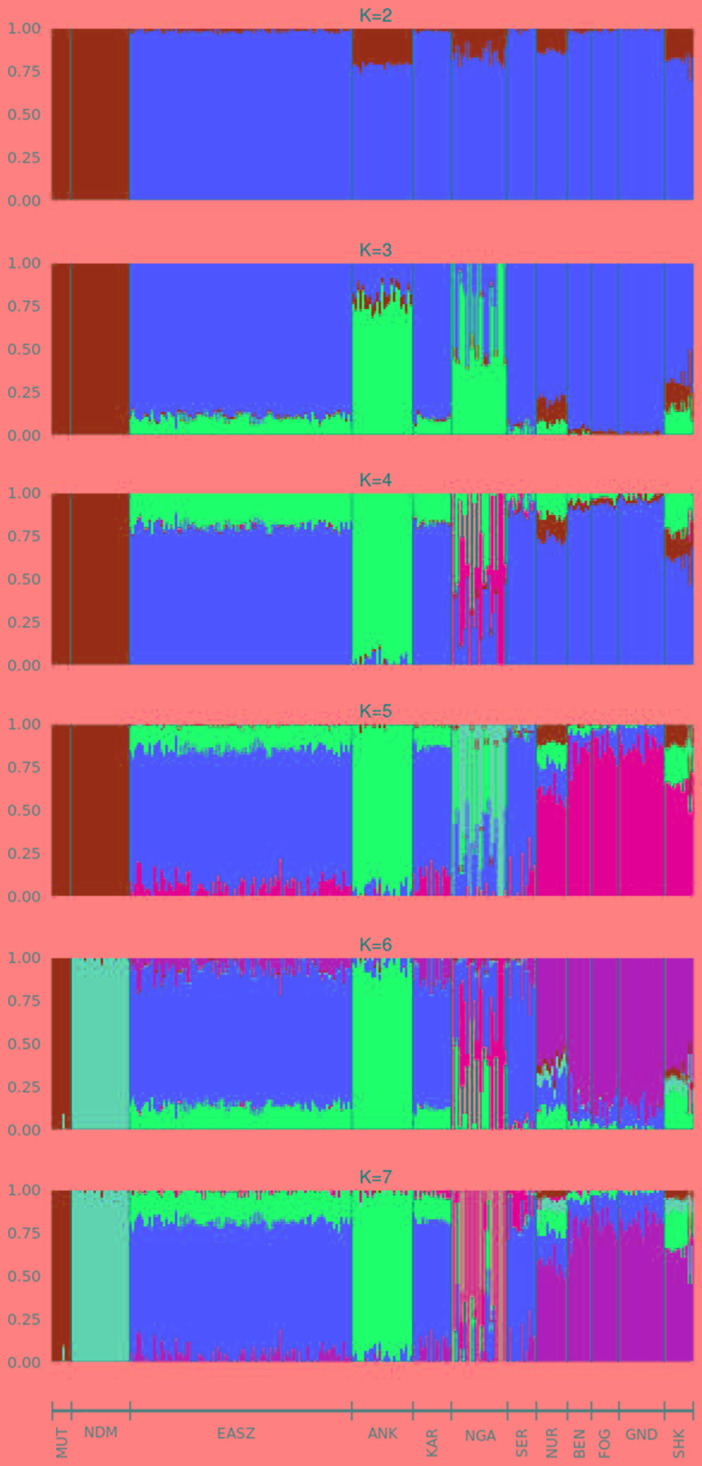
Admixture bar plots of each individual assuming different numbers of ancestral breeds (K = 2 to K = 7). ANK, Ankole; BEN, Benshangul; FOG, Fogera; GND, Gindeberet; KAR, Karamojong; MUT, Muturu; NDM, N’Dama; NGA, Nganda; NUR, Nuer; SER, Serere; SHK, Sheko.

For the further understanding of the degree of admixture in the populations, the ADMIXTURE 1.3 (Alexander et al., 2009) software was used for K = 2 to 7 hypothetical ancestral populations (**Figure 3**). We start from two hypothetical ancestral populations with the aim to determine the degree of indicine and taurine genetic background in the cattle breeds. Since the CV errors from K = 3 to K = 6 have not exceeded the cross-validation (CV) errors of K = 2, we extend the hypothetical population up to K = 7 which has the highest CV error (**Supplementary Figure 1**). At K = 2, the two ancestries taurine and indicine are revealed. The genomes of Ankole, Nganda, Nuer, and Sheko are mainly of indicine origin but have substantial taurine admixture, a result supporting our interpretation of the first PC of **Figure 2A**, that African taurine are separated from the East African indicine breeds and the mixed taurine-indicine type populations. At K = 3, Ankole, Nuer and Sheko show genetic heterogeneity with a considerable level of taurine admixture. EASZ, Karamojong, Serere, Benshangul, Fogera, and Gindeberet also show minor levels of taurine admixture whereas Nganda reveals a high level of within breed genetic differentiation. This is also in agreement with the second PC coordinate analysis in showing genetic heterogeneity within the cattle breeds (**Figure 2A**). Moreover, with the increment of the value of K, Sheko and Nuer show a higher level of genetic heterogeneity than the other east African breeds. Furthermore, at K = 6 and K = 7, the African taurine breeds N’Dama and Muturu show separate genetic backgrounds. In general, Sheko shows the highest level of African taurine genomic contribution for all values of K among East African breeds. The proportions of admixture in each of the analyzed breeds are presented for K = 7 in **Supplementary Table 1**.

Consistent with the previous findings and the origins of the genetic backgrounds of the cattle breeds worldwide (Mbole-Kariuki et al., 2014; Bahbahani et al., 2018), K = 2 highlights best the ancient divergence between indicine and taurine cattle. However, the three optimal genetic clusters suggested by the minimal CV error (**Supplementary Figure 1**) reflect the common genetic background unique to East Africa besides taurine and indicine ancestral genetic admixture. In agreement with our study, Bahbahani et al. (2018) reported east African genetic background unique to East African cattle breeds. Moreover, the admixture plots show two individuals of Sheko with a high level of taurine introgression. One of these individuals with higher taurine introgression is also detected by the PCA (**Figure 1B**, upper left corner). This could be due to the recent crossbreeding of Sheko with European dairy breeds. There were similar observations in Butana, and it was speculated that farmers might have been involved in the crossbreeding with European dairy breeds in order to increase milk production (Bahbahani et al., 2018). We believe that the introgression of the European dairy breeds into the genome of indigenous breeds such as Sheko and Butana might distort their adaptive evolutionary responses against their natural environmental stresses. In this regard, future studies should assess the impact of European dairy breeds on the genome of the indigenous African breeds with respect to their natural adaptation and tolerance attributes.

It is believed that the taurine background of the Sheko is linked to its trypanotolerance characteristics (Lemecha et al., 2006; Gibbs et al., 2009). This taurine admixture is likely a legacy of the first taurine occurrence on the African continent (Hanotte et al., 2000; Salim et al., 2014). A study on mtDNA indicates that all African cattle breeds analyzed so far carried taurine mtDNA haplotypes which suggests that these waves of indicine arrival into Africa were male-mediated (Bradley et al., 1996; Bonfiglio et al., 2012).

### Identification of Candidate Signature Genes Associated With Trypanotolerance

**Figure 4:**
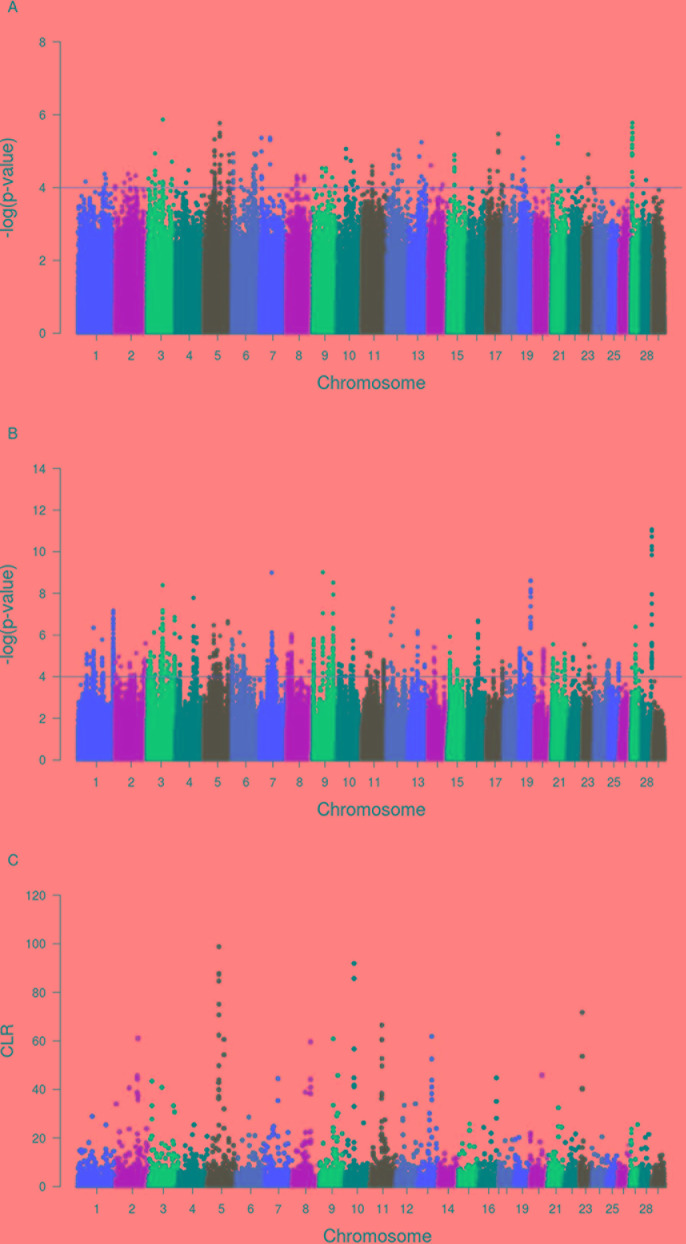
Manhattan plots of genome-wide *iHS* **(A)**, *Rsb* **(B)**, and CLR **(C)** analyses. The x-axis shows the autosomal chromosomes and the y-axis shows −log transformed *P*-values **(A** and **B)** and CLR values **(C)**.

A total of 20, 14, and 65 genomic regions harboring 109, 64, and 202 candidate signature genes were identified by *iHS*, CLR, and *Rsb* analyses in 22, 10, and 27 autosomes in Sheko, respectively (**Figure 4** and **Supplementary Tables 2**–**4**). Among the 364 unique candidate signature genes identified by *iHS*, CLR, and *Rsb* analyses, 260 disposed of enriched GO terms (α = 0.05) (**Supplementary Tables 5**–**7**). Moreover, 96, 323, and 463 intergenic variants were identified in gene desert regions by *iHS*, CLR, and *Rsb* analyses in all candidate regions, respectively (**Supplementary Tables 8**–**10**).

Mainly focusing on the top 10 candidate signature genes of each of the three methods, we performed a literature survey and identified 15 (4 genes identified by *iHS*, 3 genes identified by CLR, 7 genes identified by *Rsb*, and 1 gene identified by both *iHS* and CLR) candidate signature genes that are associated with trypanotolerant attributes which have been reported in previous studies (**Table 1**). Notably, polymorphisms in or nearby the MIGA1, CDAN1, HSPA9, and PCSK6 genes in the genome of Sheko might be associated with the evolutionary response against anemia. The MIGA1 gene is associated with iron deficiency anemia and immunity (Moura et al., 2001; Rouault, 2006). This gene also plays a major role for the development and proliferation of lymphocyte since defective T- and B-cell activation is caused by inadequate iron uptake (Rouault, 2006; Jabara et al., 2016). Another interesting candidate signature gene related with anemia is CDAN1. Polymorphisms in this gene are associated with congenital dyserythropoietic anemia type 1 (Dgany et al., 2002; Renella et al., 2011). Moreover, the hsp70 protein family and the heat shock 70kDa protein 9 (HSPA9) gene play a role as a downstream mediator of erythropoietin signaling and contribute to normal erythropoiesis (Singh et al., 1997; Ran et al., 2000; Ohtsuka et al., 2007; Chen et al., 2011). The mutation in this gene is associated with sideroblastic anemia (Schmitz-Abe et al., 2015), while the PCSK6 gene is involved in iron homeostasis and hence related with iron deficiency anemia (Guillemot and Seidah, 2015). In agreement with our findings, it has been reported by several studies that trypanotolerant N’Dama do better control anemia, a process mediated by hematopoietic cells differentiation, than trypanosusceptible breeds (Berthier et al., 2016; Naessens, 2006).

**Table 1:** Summary of major candidate signature regions identified by CLR, *iHS*, and *Rsb* analyses.

Genes	Method	CHR	Association	Position (UMD3.1) Start-End (bp)
MIGA1	Rsb	3	Anemia, immune tolerance and neurological dysfunction (Moura et al., 2001; Rouault, 2006; Jabara et al., 2016)	6706504–67137909
CDAN1	CLR	10	Anemia (Dgany et al., 2002; Renella et al., 2011)	38138863–38151656
HSPA9	Rsb	7	Anemia (Singh et al., 1997; Ran et al., 2000; Ohtsuka et al., 2007; Chen et al., 2011; Schmitz-Abe et al., 2015)	51506219–51521515
PCSK6	iHS	21	Anemia (Guillemot and Seidah, 2015)	29553201–29673109
SPAG11B	iHS	27	Immune tolerance (Yang et al., 1999; Ganz, 2003)	4920083–4942958
RAETIG	Rsb	9	Immune tolerance (Eagle and Trowsdale, 2007; Tomasec et al., 2007; Lanier, 2015)	88232044–88408862
PPP1R14C	Rsb	9	Immune tolerance, anemia and neurological dusfunction (Hanke et al., 1996; Linnekin et al., 1997; Liu et al., 2002; Haynes et al., 2003; Vignali et al., 2008)	88384683–88500749
TTC3	Rsb	1	Immune tolerance and neurological dysfunction (Chen et al., 2003; Liu et al., 2009; Pulst, 2016)	151034217–151141015
ERN1	Rsb	19	Immune tolerance and neurological dysfunction (Leach and Treacher, 1998; Oosthuyse et al., 2001; Rius et al., 2008; Liao et al., 2009; Minchenko et al., 2015; Singh et al., 2016)	48924511–48971838
CAPG	CLR	11	Immune tolerance and neurological dysfunction (Leach and Treacher, 1998; Oosthuyse et al., 2001; Rius et al., 2008; Liao et al., 2009; Zhang et al., 2009; Minchenko et al., 2015; Singh et al., 2016)	49423731–49438680
TTBK2	CRL	10	Neurological dysfunction (Jackson, 2012; Matilla-Duenas, 2012)	38159317–38248606
POLR3B	iHS	5	Neurological dysfunction (Schiffmann and van der Knaap, 2009; Daoud et al., 2013)	70062608–70178439
GNAS	iHS and CLR	13	Neurological dysfunction (Tuntasuvan et al., 1997; Bastepe, 2008; Giordani et al., 2016)	58010287–58049012
CHAT	Rsb	28	Listlessness (Johnson et al., 2016)	44143245–44187239
AP1M1	iHS	7	Listlessness (Molenaar et al., 1982)	7820650–7850254

In previous studies, trypanotolerant animals were reported to switch from innate immune response to adaptive immune response with the induction of active macrophages (M2) following trypanosome infection (Stijlemans et al., 2010; Bosschaerts et al., 2011). For instance, humoral response differences between trypanosusceptible (Boran) and trypanotolerant (N’Dama) cattle corresponding to the amount of antibody (Ab) titers have been observed. There is a difference in trypanosome-specific antiparasite Ab secreting cells in spleen and B cell activation between trypanotolerant and trypanosusceptible cattle (La Greca et al., 2014; Mamoudou et al., 2016; Morrison et al., 2016). In agreement with this, we identified the SPAG11B, RAET1G, PPP1R14C, and TTC3 genes which are involved in immune tolerance in Sheko. Interestingly, the PPP1R14C gene could play an important role in the tolerance mechanisms of Sheko with PP1, a competitive inhibitor of ATP binding of Src tyrosine kinase family members (Hanke et al., 1996; Liu et al., 2002). The inhibition of Src kinase is associated with the termination of stem cell factor induced proliferation of hemopoietic cells (Linnekin et al., 1997). It was also reported that Src kinases are involved as a primary activator of AKT (serine/threonine kinase family). AKT plays a critical role in adaptive immunity through the inhibition of regulatory T-cells (T_reg_ cells), which could play a key role in maintaining the immune tolerance (Liu et al., 2002; Haynes et al., 2003; Vignali et al., 2008). In addition, activated AKT is a mediator of neuronal cell survival (Liu et al., 2002; Chen et al., 2003; Pulst, 2016).

Furthermore, the TTC3 gene is also involved in the regulation of AKT signaling and is related with immune tolerance and neuronal cell survival (Chen et al., 2003; Liu et al., 2009; Pulst, 2016). Therefore, the mutation in the PPP1R14C gene is associated with three tolerance attributes (immune tolerance, neurological dysfunction, and anemia). Remarkably, the candidate signature gene RAET1G is one of the few genes that could encode a ligand recognized by NKG2D proteins in response to stress and infections (Eagle and Trowsdale, 2007; Tomasec et al., 2007; Lanier, 2015). Furthermore, the isoforms of the SPAG11B gene encode defensine-like peptides which are expressed by phagocytic cells (Yang et al., 1999). These structurally diverse peptides make multimeric forms during infection and disrupt the membrane of the pathogen (Ganz, 2003). They are also involved in the recruitment of T- and dendritic cells to facilitate the adaptive immunity (Yang et al., 1999). Therefore, the mutations or the differential expression of these genes are critical for the immune tolerance of Sheko to combat anemia and neurological dysfunction caused by trypanosome infection.

Trypanosomiasis is also reported to affect the nervous system of the animal. Fatihu et al. (2009) and Allam et al. (2011) reported causes of thyroid and parathyroid gland dysfunction following trypanosome infection in cattle. The dysfunctioning of thyroid and parathyroid glands often result in neurological complications or cerebral pathology (Jaggy et al., 1994; Wu and Hersh, 1994). Therefore, mutations in the POLR3B, MIGA1, TTC3, ERN1, CAPG, GNAS, and TTBK2 genes might be associated with the response to the presence of the parasite in the brain white matter, cerebral fluid, thyroid, and parathyroid glands. The endoplasmic reticulum to nucleus signaling 1 (ERN1) and capping protein gelsolin-like (CAPG) genes are involved in the regulation of hypoxia (a state of a cell with inadequate or reduced oxygen availability) (Leach and Treacher, 1998). The reduction of the hypoxic response element in the spinal cord results in the progressive degradation of the motor neuron (Oosthuyse et al., 2001; Minchenko et al., 2015). Therefore, mutations in the ERN1 and CAPG genes are associated with neurological dysfunction (Liao et al., 2009; Minchenko et al., 2015). The ERN1 and CAPG genes might also be involved in the innate immune response since hypoxia triggers innate immunity responses through the activation of the hypoxia induced factor α1 (HIF-1α) (Oosthuyse et al., 2001; Rius et al., 2008; Singh et al., 2016).

Trypanosome parasites are also known for their ability to manipulate the host immune responses. One of the mechanisms of innate immune evasion by these parasites is the reduction of HIF-1α by indolepyruvate. Therefore, the reduction of hypoxic response elements in the spinal cord results in the progressive degradation of the motor neuron (Oosthuyse et al., 2001). Therefore, the mutation in the ERN1 and CAPG genes in particular would be related to the host innate immune evasion of the parasite. Another reported candidate signature gene related with neurological dysfunction is the TTBK2 gene. A mutation in the TTBK2 gene is associated with spinocerebellar ataxia which is a genetic syndrome causing progressive degeneration of the cerebellum and the spinal cord (Jackson, 2012; Matilla-Duenas, 2012). Moreover, a mutation in the POLR3B gene is associated with hypomyelinating leukodystrophy which is characterized by a deficiency in myelin deposition of the white matter of the brain (Schiffmann and van der Knaap, 2009; Daoud et al., 2013). In addition, the POLR3B gene is also involved in positive regulation of the interferon-beta production and the innate immune response (GO:0032728, GO:0045089).

Strikingly, a mutation in the GNAS gene is associated with pseudohypoparathyroidism which is characterized by a low level of calcium and a high phosphate level in the blood (Bastepe, 2008). Allam et al. (2011) reported a similar profile during trypanosome infection in cattle that could be associated with neurological dysfunction such as muscle spasm (Tuntasuvan et al., 1997; Bastepe, 2008; Giordani et al., 2016). Furthermore, during trypanosome infection, listlessness and emaciation are some of the clinical signs of the infection (Nantulya, 1986; Steverding, 2008; Noyes et al., 2011). These clinical signs might be associated with the destruction of the thyroid gland by trypanosome parasites in cattle (Fatihu et al., 2009). The candidate signature genes AP1M1 and CHAT are related with these clinical signs. Most importantly, the AP1M1 gene is a member of the adapter protein complex which is involved in thyroid abnormalities (Johnson et al., 2016). Due to the thyroid gland dysfunction (hypothyroidism), the nerves are unable to conduct electrical impulses properly. This leads to general weakness, lethargy, and listlessness (Jaggy et al., 1994). The mutation in the CHAT gene is associated with myasthenia gravis which is an autoimmune disease characterized by load dependent muscle weakness (Molenaar et al., 1982).

**Figure 5:**
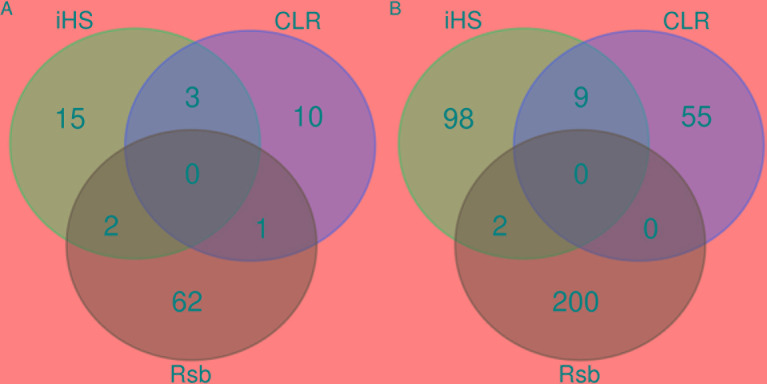
Venn diagrams of the overlapping **(A)** genomic regions and **(B)** candidate genes identified by *iHS*, CLR, and *Rsb*.

Our findings show strong selective sweeps (**Figures 4A–C**) in the genomic regions around the selected signature genes of **Table 1** (**Supplementary Table 11**). This might indicate that the mutations in these genes have reached fixation or are near fixation. Therefore, the identified candidate signature genes in **Table 1** might play a major role in the natural tolerance attributes of Sheko against trypanosomiasis. Moreover, the comparison of candidate signature genes identified by the *iHS*, CLR, and *Rsb* methods show more overlaps between *iHS* and CLR than between *Rsb* and *iHS* or CLR analyses (**Figures 5A, B**), in agreement with *Rsb* being a powerful method to detect selection signature when the selected allele has reached fixation (Tang et al., 2007; Oleksyk et al., 2010; Bahbahani et al., 2018).

Among the 15 identified candidate signature genes (**Table 1**), the MIGA1, RAETG, and PPP1R1AC genes are not significantly functionally enriched (α = 0.05). This might indicate that these candidate signature genes in Sheko could be specific to the environmental pressure in the region such as trypanosomiasis. Moreover, the identified signature regions of the three methods were compared with trypanotolerant QTL regions which were reported by Hanotte et al. (2003). Among the 55 trypanotolerant QTL, which were identified by crossing trypanotolerant N’Dama and susceptible Boran, 6 regions were overlapping with trypanotolerant QTL in N’Dama (**Supplementary Table 12**). Interestingly, among the identified candidate signature genes in **Table 1**, the AP1M1 and GNAS genes are found in these overlapping regions. The overlapping regions and genes of Sheko and N’Dama might indicate occurrence of selection at the same genes in these two breeds against the same environmental pressures.

### Functional Annotation of Candidate Signature Genes

**Figure 6:**
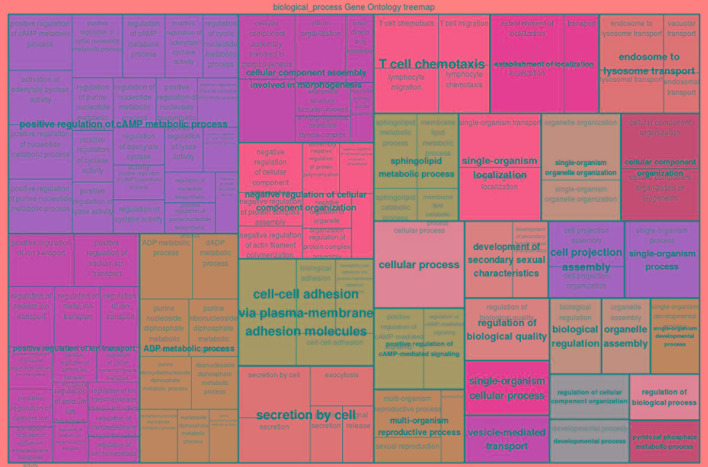
GO treemap for the 260 functionally enriched (*P* < 0.05) genes. The size of the boxes corresponds to the −log10 *P*-value of the GO-term. The boxes are grouped together based on the upper-hierarchy GO-term which is written in bold letters.

In order to characterize the biological functions of functionally enriched candidate genes, a treemap was produced using the geneXplain platform (Krull et al., 2006). The treemap shows the clusters of 30 functional terms. Most of these terms are associated with cellular transport, metabolic processes and biological regulation (**Figure 6**). Interestingly, among the 30 enriched terms, two GO-terms are T-cell chemotaxis and cell–cell adhesion which play a critical role in the immune system (Springer, 1990; Gerard and Rollins, 2001; Bach et al., 2007). T-cell chemotaxis (chemoattractant cytokines) is a process that requires the movement of T-cells in response to a certain signal or external stimulus. The movement or circulation of immune cells in the blood and lymph as non-adherent cells and in tissues as adherent cells is critical for patrolling the body against infectious organisms effectively (Springer, 1990). For instance, β defensin is chemotactic for chemokine receptors of macrophages, natural killer cells, immature dendritic cells, and memory T-cells. Therefore, the recruitment of these cells to the site of a microbial invasion provides a link between innate and adaptive immunity (Yang et al., 1999). Likewise, T-cell mediated migration of thymocyte toward chemokines was observed following trypanosome infection in human (Mendes-da Cruz et al., 2006). In the presence of infectious organisms (foreign antigens), the immune cells aggregate at the site of the infection and through their adhesion receptors they adhere to cells bearing a foreign antigen (Springer, 1990).

### Identification of Overrepresented Pathways in the Candidate Signature Gene Sets

Pathway analysis has become a powerful tool in order to refine the molecular mechanisms of disease tolerance. The rationale of pathway analysis lies in the detection of overrepresentation of biologically defined pathways based upon the functionally enriched candidate selected genes. We performed pathway analysis using the TRANSPATH database on the geneXplain platform. The TRANSPATH pathway analysis identified 15 genes out of 260 functionally enriched genes that are involved in 13 overrepresented TRANSPATH pathways (**Table 2**). Among these genes, the immunoproteasome PSMD7 gene is involved in most of the overrepresented pathways. This gene is involved in the processes of presenting antigens by the major histocompatibility complex (MHC) class I proteins to CD8+ T-lymphocytes (Morrot and Zavala, 2004; Goldszmid and Sher, 2010; Jordan and Hunter, 2010). Sufficient induction of CD8+ during infection leads to pathogen elimination. It has been reported that immunoproteasome subunits are key determinants of the CD8+ T-cell level and quality involved in host resistance to trypanosomes infection (Ersching et al., 2016). This gene plays a critical role in the development of adaptive immunity or tolerance (Doolan and Hoffman, 1999).

**Table 2:** Overrepresented pathways for the identified candidate signature genes.

Pathway	Raw *P*-value	Genes
PDGF B —> STATs	0.003	STAT3, STAT5A
Stress-associated pathways	0.007	MBP, MEF2A, PSMD7, RAF1, RBX1, STAT3
E2F network	0.008	AKT3, CDC25C, PPP2R5A, PSMD7, RAF1, RBX1
G2/M phase (cyclin B:Cdk1)	0.015	AKT3, CDC25C, PSMD7, RBX1
IMP —> ADP	0.025	AK5, AMPD3
ARIP1 —> atrophin1	0.034	AKT3, APBA1
p38 pathway	0.039	MBP, MEF2A, STAT3
Plk1 cell cycle regulation	0.039	CDC25C, PSMD7, RBX1
IL-3 signaling	0.043	MBP, RAF1, STAT5A
Aurora-B cell cycle regulation	0.045	CENPE, PSMD7, RBX1
Oxygen independent HIF-1alpha degradation	0.045	PSMD7, RBX1, UBE2R2
Cul3 —/Nrf2	0.047	PSMD7, RBX1
S phase (Cdk2)	0.048	CDC25C, RAF1, RBX1

The names of the pathways are provided by the TRANSPATH database on the geneXplain platform.

However, adaptive immunity also plays a key role for the emergence of auto-immunity. Previous studies indicate that trypanosome infection could deplete thymocytes. As a result, immature T-lymphocytes are released from the thymic central tolerance and differentiate into mature T-helper cells in the lymph nodes (Flávia Nardy et al., 2015). This process would induce auto-immunity against self-antigens. Moreover, during trypanosome infection, the red blood cell membrane might be damaged by parasite enzymes such as proteases or phospholipases. This could expose epitopes which are not recognized as self-antigens and would trigger immune-mediated hemolysis due to antibody response against these self-antigens (Taylor, 1998). This could be controlled by suppressing the development of auto-reactive immune cells through ubiquitination which is a degradative tag to be recognized by a proteasome complex such as PSMD7 (Lodish et al., 2004; Zinngrebe et al., 2014). Furthermore, some of the identified candidate signature genes are also associated with protein ubiquitination processes which might indicate that these genes are also involved in the functions described above (**Supplementary Tables 2**–**4**). To the best of our knowledge, our study is the first to show the potential of a molecular mechanism for controlling auto-reactive immune cells caused by trypanosomiasis in cattle. In agreement with our finding, Kierstein et al. (2006) reported that a trypanotolerant mouse strain showed overexpression of several genes encoding proteases.

In general, most of the overrepresented pathways (PDGFB –> STATs, stress associated pathways, IMP –> ADP, ARIP1 –> atrophin 1, p38 pathway, IL-3 signaling, oxygen independent HIF-1alpha degradiation and Cul3 –/Nrf2) pathways are activated by cellular stresses and antigens while others [E2F network, G2/M phase (cyclin B:Cdk1), S phase (Cdk2), Plk1 cell cycle regulation and Aurora-B cell cycle regulation] pathways are involved in cell cycle processes.

The first two pathways in **Table 2** (PDGFB –> STATs and stress associated pathways) are related to the immune system and anemia. Especially, in stress associated pathways we find MBP, RAF1, MEF2A, and STAT3 genes that are involved in the immune and nervous systems. In the MBP gene, there are eight different mRNAs due to alternative splicing of exons (Zelenika et al., 1993). Three of the eight splice variants are expressed in the brain, macrophages and hemolymphopoietic tissues such as spleen, bone marrow, and thymus (Zelenika et al., 1993). This gene is also involved in the interleukin (IL)-3 signaling pathway. IL-3 is a T-cell-derived hematopoiesis stimulating cytokine involved in the production, differentiation and function of granulocytes and macrophages (Ymer et al., 1985; Dorssers et al., 1987). This suggested that the expression of alternatively spliced MBP mRNAs is related with the immune system in response to trypansome infection or the presence of a pathogen in the central nervous system. The serine/threonine kinase proto-oncogene RAF1 is also related with the stress associated pathway and is involved in inducing adaptive immunity by regulating the expression of cytokines that are important for the differentiation of T-helper cells (Gringhuis et al., 2009).

Moreover, STATs family members are also involved in the activation of various cytokines and in the promotion of cell survival by inducing the expression of antiapoptotic BCL2L1/BCL-X(L) genes (Benito et al., 1996; Packham et al., 1998; Yuan et al., 2004). For instance, STAT3 activation by trypomastigotes was associated with the survival of cardiomyocytes during infection (Ponce et al., 2012; Stahl et al., 2013). The other gene involved in defense response is MEF2A which is associated with promoting antimicrobial peptide expression during infection (Clark et al., 2013). This gene is also involved in neuronal cell survival and loss of function (Gong et al., 2003; She et al., 2011). As reported by She et al. (2012), neurotoxins induce ubiquitination of MEF2A in response to toxic stress which leads to the loss of neuronal viability. Furthermore, He et al. (2015) reported that increased platelet-derived growth factor (PDGF)-B related signaling is associated with induced chemokine secretion which is a mediator of innate and adaptive immune responses (Kim and Broxmeyer, 1999). In addition, knock-out mice for PDGF-B develop anemia (Kaminski et al., 2001) which indicates that the PDGFB –> STATs pathway is also involved in this disease.

The E2F network as well as the Cdk1 and Cdk2 related pathways are also associated with anemia which is the most prominent and consistent clinical sign of trypanosome infection (Kaminski et al., 2001; Dimova and Dyson, 2005; Noyes et al., 2011, Hu and Sun, 2016). The tumor suppressor retinoblastoma (Rb) is the inhibitor of E2Fs. When Rb binds to E2Fs, it prevents E2F mediated activation of transcriptional genes. In quiescent cells, E2F is required for the cell differentiation through a series of signal transduction cascades, including Cdks activation and phosphorylation. The Aurora-B and Plk1 pathways are involved in the activation and phosphorylation of Cdks, respectively. As a result of these and several other signaling cascades, E2Fs is activated while inactivating Rb. The activated E2F mediates quiescent cells for S phase entry and cell cycle progression (Dyson, 1998; Nevins, 1998; Trimarchi and Lees, 2002; Dimova and Dyson, 2005;Song et al., 2007). Hu et al. (2012) reported that mice deficient for both E2F8 (i.e., E2F gene family) and Rb show severe anemia.

Furthermore, the hypoxia inducible factor (HIF) and the nuclear factor-erythroid 2-related factor 2 (NRF2) pathways are related with anemia (Lee et al., 2004;Silva and Faustino, 2015). During hypoxia, HIF facilitates a high production of red blood cell (erythropoiesis) in order to overcome shortage of oxygen (Silva and Faustino, 2015). The other pathway, NRF2, regulates the expression of antioxidant responsive element-driven genes and plays a critical role in the antioxidant responsive element-driven cellular protection (Cho et al., 2002). In addition, knockout mice for NRF2 show regenerative immune-mediated hemolytic anemia which indicates that this pathway is involved in erythrocyte maintenance during oxidative stress (Lee et al., 2004).

Intriguingly, serine/threonine kinase family isoforms of the AKT gene are involved in the E2F, Cdk1, IMP-ADP, and ARIP1-atrophin1 pathways. This gene is activated in the host cells during trypanosome infection (Woolsey et al., 2003; Chuenkova and PereiraPerrin, 2009). The host kinase AKT promotes infected host cell survival and restricts the growth of intracellular parasites (Caradonna et al., 2013). AKT3 is also a key mediator of down stream signaling pathways of activated receptor tyrosine kinases which play a role in STAT3 activation (Yuan et al., 2004; Chuenkova and PereiraPerrin, 2009. The different isoforms of the kinase AKT regulate the development of immunity and autoimmuniy. Zhang et al. (2013) reported that AKT is predominantly expressed in the innate immune cells. The isoforms of AKT are primarily involved in regulating inflammatory responses although it has been reported that AKT also modulates adaptive immune responses (Liu et al., 2002).

Moreover, the AKT related pathway Atrophin-1 plays a role in erythroid and lymphoid cell differentiation and in E3 ubiquitin ligase atrophin-1 interacting protein 4 (ITCH) signaling cascades. Atrophin-1 is involved in the regulation of immune responses through Notch-mediated signaling pathways (Qiu et al., 2000; You et al., 2009; Aki et al., 2015). It is also associated with spinocerebellar degeneration caused by extended CAG repeats encoding several glutamine units (polyglutamine tract) in the atrophin-1 protein (Kanazawa, 1999). The disease is characterized by neurological symptoms such as ataxia which is one of the clinical signs of trypanosome infection (Tuntasuvan et al., 1997; Suzuki and Yazawa, 2011; Giordani et al., 2016).

Further important pathways are p38, IMP –> ADP, and the aurora B-cell cycle regulation pathways that are involved in the host defense mechanism. The p38 pathway is a MAPK-related pathway which is activated by various physical and chemical stresses, such as hypoxia and various cytokines. The activation of the p38 pathway is critical for normal immunity and inflammatory responses (Roux and Blenis, 2004). Moreover, the AK5 and AMPD3 genes are involved in the IMP –> ADP pathway and play a central role in the regulation of inflammation and red blood cell homeostasis (Tavazzi et al., 2000; Mabley and Szabo, 2008). AK5 is associated with double positive thymocyte and auto-immunity regulation in the brain and pancreatic tissues (Stanojevic et al., 2008) while the AMPD3 gene is involved in the regulation of the energy state of red blood cells during oxidative stress (hypoxia) (Tavazzi et al., 2000). In addition to that, the aurora B-cell cycle regulation pathway is involved in the progression of T-lymphocytes which play a critical role for the development of innate and adaptive immunity (Song et al., 2007; Paul et al., 2011). To this end, the HIF and NRF2 related pathways are directly associated with the induction of host innate and adaptive immunity under oxidative stress (Singh et al., 1997; Cramer et al., 2003; Jantsch et al., 2011; McNamee et al., 2013; Battino et al., 2018).

In summary, our findings of the search for signature genes appear to be well substantiated by the results of the overrepresented pathways analysis. This implies that most of the overrepresented pathways are mainly associated with host defense mechanisms against pathogens and anemia. Particularly, stress-associated, HIF and NRF2 related pathways are involved in oxidative stress responses. Interestingly, trypanosome infection induces the production of superoxide, hydrogen peroxide, peroxyl radicals, and hydroxyl radicals which are known to cause oxidative stress followed by tissue damage and hemolysis (Saleh et al., 2009). Under oxidative stress (hypoxia), erythrocytes are important mobile oxidative sinks (antioxidant) for themselves, other cells, and tissues. However, these properties of the red blood cells during oxidative stress contribute to its susceptibility toward hemolysis which leads to anemia (Chan et al., 2001; Sangokoya et al., 2010). In order to overcome the shortage of oxygen, stress-associated, HIF, and NRF2 related pathways play a critical role in the production of red blood cells in which hemoglobin acts as oxygen repository for red blood cells and other cells (Chan et al., 2001; Sangokoya et al., 2010; Silva and Faustino, 2015).

None of the most significant candidate signature genes (**Table 1**) was contained in the overrepresented pathway gene list (**Table 2**). This indicates that the candidate signature genes might be involved in the evolutionary gear particularly toward trypanotolerance in Sheko. For instance, candidate signature genes involved in the regulation of hypoxia (ERN1 and CAPG) are not identified in the overrepresented hypoxia related pathways. This might indicate that these candidate signature genes might be specific to oxidative stress tolerance attributes in Sheko. Hence, trypanotolerance of Sheko could be controlled by some major selected genes whose major effect close to fixation in the breed (become breed characteristic) and cohorts of genes with minor effects.

### Identification of Master Regulators Based on Candidate Signature Genes

**Figure 7:**
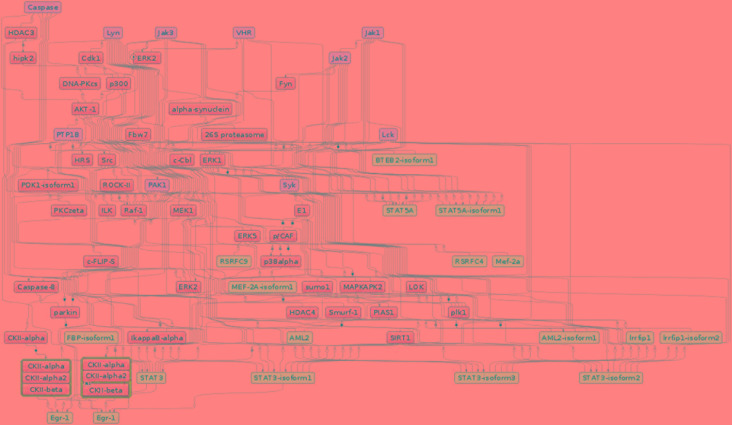
The master regulatory networks for Sheko (Caspase, Lyn, Jak1, Jak2, Jak3, VHR, PTP1B, PAK1, Lck, and Syk). Red, blue, and green indicate master regulators, regulated proteins, and connecting molecules, respectively.

To gain more insight into the regulatory mechanisms of the identified candidate signature genes, we performed a master regulatory network analysis using the TRANSPATH database in the geneXplain platform. Applying the maximum radius of 10 steps upstream in the regulatory hierarchy, we identified ten master regulators (**Figure 7**). Remarkably, the master regulator Caspase, which is a family of protease enzymes, is associated mainly with regulating the reduction of the load of intracellular parasites, induction of nitric oxide production, increasing the level of CD4 and CD8+ T-cells, secretion of IFNγ, and control of trypanosome infection by macrophages (Gonçalves et al., 2013). This master regulator is involved in programmed cell death such as pyroptosis and necroptosis. These types of programmed cell deaths play a role for protecting an organism against oxidative stress (stress signals) and pathogenic attack (Shalini et al., 2015). In addition, Caspase also plays a role in the normal erythroid differentiation in the terminal stages (Zermati et al., 2001).

Most of the regulatory molecules (Syk, Lck, Lyn, Jak1, Jak2, and Jak3) are protein tyrosine kinases while others (VHR and PTP1B) are protein tyrosine phosphatases and activated kinase (PAK1). These master regulators are mainly associated with innate and adaptive immune responses and are critical for the functioning of the nervous and immune systems. For instance, the activation of the regulatory molecule Syk requires the regulatory molecule Lck to phosphorylate immunoreceptor tyrosine-based activation motifs. Then, the phosphorylated immunoreceptor tyrosine-based activation motif modulates T-cell proliferation and differentiation by recruiting Syk protein tyrosine kinases (Acuto et al., 2008; Au-Yeung et al., 2009). In addition, coupling of the other master molecules JAK1 and JAK3 occurs on the cell surface receptor of IFNγ, followed by phosphorylation of the IFNγ receptor 1. This process leads to the activation of the STAT1 protein. The STAT1 protein binds to the target element of the IFNγ inducible gene in the nucleus and facilitates the transcription of the target regions during immunity responses (Rosenzweig and Holland, 2005; Casanova and Abel, 2007). Another reported regulator molecule VHR is also involved in the phosphorylation of STAT proteins and in the T-lymphocyte physiology (Alonso et al., 2001; Hoyt et al., 2007). Moreover, the master molecule JAK2 plays a critical role in the maintenance of hematopoiesis. It has been shown that selective deletion of JAK2 results in lethal anemia in adult mice (Grisouard et al., 2014).

Furthermore, a related master molecule, the protein tyrosine phosphatase 1B (PTP1B), is reported to modulate the activation of macrophages and plays a key role in mediating the central dendritic cell function of bridging innate and adaptive immunity (Heinonen et al., 2006; Martin-Granados et al., 2015). The kinase family regulator molecule Lyn is also involved in the regulation of innate and adaptive immune responses (Ingley, 2012). Lyn is also known for mediating the production of type I interferone (IFN-I) which is involved in host defense mechanisms against invading pathogens (Kawai and Akira, 2007; Blasius and Beutler, 2010; McNab et al., 2015). The related kinase regulatory molecule PAK1 is highly expressed in most leukocytes that are involved in immune responses. PAK1 also plays an important role in the activation of MAP-kinase pathways which are involved in all aspects of immune responses, from innate immunity to the activation of adaptive immune responses (Yi et al., 1991; Adachi et al., 1992; Zhang et al., 1995; Dong et al., 2002; Wang et al., 2002; Traves et al., 2014). In general, these proteins and master regulatory molecules are a large family of signaling enzymes that are expressed in various immune cells and regulate immune cell differentiation, cytokine production, and immune responses. Therefore, to maintain the tolerance against a pathogen, the regulation of these signaling pathways is critical (Manning et al., 2002; Salmond et al., 2009).

Strikingly, stress-induced protein kinases could also induce or aggravate auto-immunity by phosphorylating self-antigens to be recognized by auto-antibodies (Utz et al., 1997; Patterson et al., 2014). However, Caspase-mediated apoptosis plays an important role in arresting the development of auto-immunity by eliminating auto-reactive and pro-inflammatory cells (Eguchi, 2001). Moreover, the activation of Caspase and JAK2 is essential for the processes of erythroid differentiation and for the maintenance of hematopoiesis (Zermati et al., 2001). On the other hand, the inhibition of Caspase dependent mechanisms contributes to cell survival (Lamkanfi et al., 2007). We believe that the candidate signature genes involved in anemia, neurological dysfunction, listlessness, and immune tolerance might be governed by the top master regulator Caspase in harmony with other regulatory molecules. In general, our study provides a first report on the top master regulators for trypanotolerance of Sheko and the overall analysis framework might be helpful to understand the underlying mechanisms of different cattle diseases in future works.

## Materials and Methods

### SNP Genotyping and Quality Control

sDNA was extracted from 67 blood and tissue samples according to the QIAGEN DNA extraction protocol (**Supplementary Table 13**). 19 samples from Gindeberet, 12 from Sheko, 13 from Nuer, 12 from Benshangul and 11 from Fogera breeds were collected. All samples were taken randomly from unrelated animals based on the information given by livestock keepers at the time of sampling. All samples were genotyped for 777,962 SNPs using the Illumina BovineHD Genotyping Bead chip. In addition, the genotyping data of two west African breeds (24 N’Dama and 8 Muturu), and five east African breeds (92 EASZ, 25 Ankole, 16 Karamojong, 23 Nganda, and 12 Serere) were obtained from the International Livestock Reaserch Institute (ILRI, Addis Ababa, Ethiopia; Bahbahani et al. (2017)). For quality control, Plink1.9 (Purcell et al., 2007) was used on 735,293 autosomal SNPs. SNPs with minor allele frequency of less than 1% were excluded (19,581 SNPs). Minimum genotyping call rate (<95%) and maximum identity-by-state (IBS) (≥95%) were also used as filtering criteria. Two Benshangul samples failed the genotyping call rate criteria and were excluded from the analysis but no pair of samples was excluded due to the IBS filtering criterion. The total sample size for the down stream analysis consisted of 265 samples and 715,712 SNPs. BEAGLE 4 (Browning and Browning, 2007) was used for inferring haplotype phasing and imputing the missing alleles. The imputation was performed by fitting 83 sliding windows across the autosomes in which on average 8600 markers were included. With in each window 12 iterations were executed. Since our samples consist of indigenous African breeds, the total of 264 (n − 1) animals included in this study are used as a background to impute the missing alleles in the context of indigenous African cattle genome (i.e., without using the reference genome).

### Genetic Background of the Cattle Population

In the eastern part of Africa, the mixture of African taurine and indicine cattle populations is common which reflects the wave of these two different ancestral aurochs in the region (Hanotte et al., 2000; Salim et al., 2014; Bahbahani et al., 2017). Regarding these two ancestral populations, the N’Dama and Muturu breeds are considered as African taurine whereas the Fogera, EASZ, Ankole, Karamojong, and Serere breeds are referred to as African zebu (Bahbahani et al., 2017). The Nuer and Ankole breeds are classified as African sanga (DAGRIS, 2007) while the Nganda breed is assigned to African zenga (Bahbahani et al., 2017). The sanga and zenga cattle are crossbreds between the indigenous humpless cattle and zebu. The latter have higher zebu genetic introgression than the former (Rege, 1999). Interestingly, the Sheko breed is considered as the last oddments of the primordial *Bos taurus* cattle in eastern Africa. However, some animals in the present population of Sheko display small humps which indicates the genetic introgression of zebu cattle (DAGRIS, 2007). Yet, there is no research publication or documentation available on the genetic background of the Benshangul and Gindeberet breeds which are included in this study. The breed type and origin of the cattle samples included in this study are presented in **Table 3**.

**Table 3:** Cattle breeds included in the study.

Breed name	Breed category*	Breed origin
N’Dama	African taurine	Guinea
Muturu	African taurine	Nigeria
Ankole	Sanga	Uganda
Karamojong	African zebu	Uganda
Serere	African zebu	Uganda
Nganda	Zenga	Uganda
EASZ	African zebu	Kenya
Sheko	African taurine and zebu	Ethiopia
Nuer	Sanga	Ethiopia
Gindeberet	Not available	Ethiopia
Benshangul	Not available	Ethiopia
Fogera	African zebu	Ethiopia

*Breed category according to DAGRIS (2007).

EASZ, East African Shorthorn Zebu.

### Breed Differentiation, Genetic Relationship, and Structure

In order to understand the genomic structure of Sheko, we considered in total 12 indigenous African breeds genotyped with the Illumina BovineHD Genotyping BeadChip. To assess the within and between population genetic structure and admixture, PCA and admixture analyses were conducted. PCA was performed using Plink 1.9 to estimate the eigenvectors of the variance-standardized relationship matrix of all samples. In order to refine the genetic structure of the indigenous Ethiopian cattle breeds, separate PCA calculation were made for samples that were collected in Ethiopia (Sheko, Benshangul, Gindeberet, Fogera, and Nuer). Admixture analysis was performed using the ADMIXTURE 1.3 software with CV and 200 bootstraps for the hypothetical number of ancestries K (2 ⩽ K ⩽ 7). Both PCA and admixture analyses were used to determine the level of admixture and genetic differentiation of the populations. Furthermore, admixture analysis was used to determine the level of indicine and taurine ancestries of each breed at the genome-wide level. In particular, PCA and admixture analyses were performed to show the taurine background of Sheko.

### Analysis of Signatures of Positive Selection

In general, methods for the detection of selection signatures are based on the spatial distribution of allele frequencies and the property of segregating haplotypes in the population (Hayes et al., 2010). As suggested by Ma et al. (2015) and Vatsiou et al. (2016), combining these methods would help to reach a higher power than with single analysis. In this paper, we used EHH and spatial distribution of allele frequency-based methods to identify signatures of positive selection in the genome of the Sheko breed. This denotes that integrated haplotype score (*iHS*) and CLR analyses were performed on Sheko (12) while the ratio of site-specific EHH (EHHS) between populations (*Rsb*) analysis were performed between Sheko (12) and combined trypanosusceptible reference cattle populations (179) [EASZ (92) (Muhanguzi et al., 2014; Van Wyk et al., 2014), Ankole (25) (Magona et al., 2004), Karamojong (16) (Muhanguzi et al., 2017), Nganda (23) (FAO, 2004), Serere (12) (Ocaido et al., 2005) and Fogera (11) (Sinshaw et al., 2006)]. The results of these tests were combined into one gene set.

#### Extended Haplotype Homozygosity Based Methods

*Rsb* and *iHS* are LD based approaches which are implemented in R package *rehh*. Both *Rsb* and *iHS* are used to identify genome-wide signatures of selection (Gautier and Vitalis, 2012). These tests start with a core haplotype (i.e., a set of closely linked SNPs in which recombination does not take place) identification (Sabeti et al., 2002; Skipper, 2002). Then, the decay of LD as a function of the distance from the core haplotypes is analyzed (Sabeti et al., 2002). The *Rsb* analysis was performed between Sheko and the combined group of trypanosusceptible breeds. For each group, integrated site-specific EHH of each SNP (iES) was calculated. Standardized log-ratio between iES of the two groups was used to calculate *Rsb* values. The *iHS* values were calculated for Sheko as the natural log ratio of integrated EHH (iHH) between reference and alternative alleles for each SNP (Gautier and Vitalis, 2012; Bahbahani et al., 2018). The bovine reference genome (UMD3.1) is used as the reference allele while the study population (Sheko) is considered as the alternative allele. The *iHS* values were standardized based on the calculated mean and standard deviation values. This allows direct comparisons among different SNPs regardless of their allele frequencies (Gautier and Vitalis, 2012). For the standardization of *Rsb* values, median and standard deviation values were used. One-tailed Z-tests for *Rsb* and two-tailed Z-tests for *iHS* were applied on the standardized and normally distributed *Rsb* and *iHS* values (**Supplementary Figures 2A, B**) to identify statistically significant SNPs that are under positive selection. For one-tailed Z-tests, *P* = 1 − Φ(Rsb), whereas *P* = 1 − 2|Φ(iHS) − 0.5| was used for the two sided tests with Φ being the Gaussian cumulative density function. For both *Rsb* and *iHS P*-values, the significance threshold of α = 10^−4^ was applied following the study of Bahbahani et al. (2018) to identify candidate regions.

#### Spatial Distribution of Allele Frequency Based Method

The CLR test is an LD based selective sweep searching algorithms using the information from the spatial distribution of allele frequencies (Charlesworth, 2012). CLR is used to identify skewed patterns of the allele frequency spectrum toward excess of rare alleles and high frequency alternative alleles due to the hitchhiking effect (Kim and Stephan, 2002; Nielsen et al., 2005; Qanbari et al., 2014). The *P*-values were calculated by the rank of the genome wide scan of CLR values. As suggested by Wilches et al.(2014), the 95^th^) quantile of the distribution of the top CLR *P*-values was used to identify a significance threshold of α=10^-5^ (**Supplementary Figure 3**). For CLR analysis, the Sweepfinder2 (DeGiorgio et al., 2016) software was used for each chromosome with a window size of 50kb including on average 226 SNPs per window. Sweepfinder2 estimates CLRs in the context of background selection to identify sweeps (DeGiorgio et al., 2016; Huber et al., 2016).

### Functional Annotation of Selected Candidate Regions

Genes found within 25 kb around the most significant SNP were considered as candidate genes (Bahbahani et al., 2018). Protein-coding and RNA genes found within the candidate regions were retrieved using the BioMart tool (Kinsella et al., 2011). The R package *Enrichr* (Kuleshov et al., 2016) was used to determine the candidate signature genes that are functionally enriched in GO terms with respect to the whole bovine reference genome background (α = 0.05). These functionally enriched candidate signature genes were used to produce a treemap which shows clusters of functional terms based on the biological functions of the candidate signature genes.

To gain more insight into the functional properties and molecular mechanisms involved in trypanotolerance, overrepresented pathways were analyzed using the TRANSPATH database (Krull et al., 2006) of the geneXplain platform (http://genexplain.com/). Furthermore, to understand the regulatory mechanisms of the candidate signature genes and the signaling cascades in the regulatory hierarchy involved in trypanotolerance, the identification of master regulators was conducted using the TRANSPATH database.

## Conclusion

For generations, African animal trypansomiasis has been the major selection pressure in the region. We have identified the candidate causative genes, pathways, and master regulators associated with the adaptation of the Sheko breed to its natural environmental pressure. Most of the identified candidate signature genes, overrepresented pathways, and master regulator molecules were involved in immune tolerance, neurological dysfunction, and anemia. This entails that the genome of Sheko was targeted by these environmental pressures which are associated with trypanosomiasis. Therefore, this study helps as an input for designing and implementing genetic intervention strategies to improve the performance of susceptible as well as animals which are relatively tolerant toward higher trypanotolerance. The improvement of the cattle health contributes to increase the production of milk and meat. The improvement of the cattle health enhances the draft power of the animal which is associated with increasing crop production. This implies that, increasing animal and crop production significantly contributes to eradicate poverty in the area. In general, this study contributes to the existing literature in two ways: 1) The genetic controls of Sheko against trypanosomiasis have not been well studied and this study examines the genomic signatures in response to trypanosomiasis in detail; 2) this study presents pathways and master regulators which could help to understand the upstream biological processes involved in trypanotolerance. Particularly, this study for the first time identifies the master regulators involved in the regulatory mechanisms of trypanotolerance in relation to signatures of selection not only for Sheko breed but also in the context of cattle genomics, which can be used for the development of effective new drugs. However, additional studies such as differential expressions of targeted genes and regulatory molecules may be required to further confirm the validity of the results reported in this paper.

## Data Availability Statement

The SNP data in this study can be found in the European Variation Archive (EVA): PRJEB34751.

## Ethics Statement

Standard techniques were used to collect blood. The procedure was reviewed and approved by the University of Edinburgh Ethics Committee (reference number OS 03-06) and also by the Institute Animal Care and Use Committee of the International Livestock Research Institute, Nairobi.

## Author Contributions

YM, MG, and AS participated in the design of the study. YM conducted computational and statistical analyses as well as identified the signature genes. AS and MG supervised the computational and statistical analyses. YM interpreted the results with MG. YM carried out the literature survey and prepared the first draft of the manuscript. OH and KE were involved in the interpretation of the results. YM and KE were involved in collecting blood and tissue samples for this study. YM prepared the DNA samples. YM and AS were involved in the preparation of the genotyping data. YM and MG wrote the final version of the manuscript. YM, AS, and MG conceived and managed the project. All authors read and approved the final manuscript.

## Conflict of Interest

The authors declare that the research was conducted in the absence of any commercial or financial relationships that could be construed as a potential conflict of interest.

## Supplementary Material

Supplementary Figure 1 Cross validation error in relation to the number of hypothetical ancestral populations for the Admixture analyses.

Supplementary Figure 2 Histogram of standardized *Rsb* and *iHS* values.

Supplementary Figure 3 Box plot of CLR -log (*P*-values).

Supplementary Table 1 Proportion of admixture within each of the analyzed breeds.

Supplementary Table 2 Functionally annotated gene list identified by *iHS* analysis.

Supplementary Table 3 Functionally annotated gene list identified by CLR analysis.

Supplementary Table 4 Functionally annotated gene list identified by *Rsb* analysis.

Supplementary Table 5 Functionally enriched gene list identified by *iHS* analysis.

Supplementary Table 6 Functionally enriched gene list identified by CLR analysis.

Supplementary Table 7 Functionally enriched gene list identified by *Rsb* analysis.

Supplementary Table 8 List of intergenic variants identified by *iHS* analysis.

Supplementary Table 9 List of intergenic variants identified by CLR analysis.

Supplementary Table 10 List of intergenic variants identified by *Rsb* analysis.

Supplementary Table 11 Summary of the genomic regions identified by *iHS*, CLR and *Rsb*.

Supplementary Table 12 QTL regions overlapping between Sheko and N’Dama.

Supplementary Table 13 Summary of the blood and tissue samples collected from indigenous Ethiopian cattle breeds for DNA extraction.
