# Supplementary figures and images for: Identification of Candidate Signature Genes and Key Regulators Associated With Trypanotolerance in the Sheko Breed
Source: Front Genet. 2019 Nov 14;10:1095. doi: 10.3389/fgene.2019.01095 (PMC6872528; doi:10.3389/fgene.2019.01095)

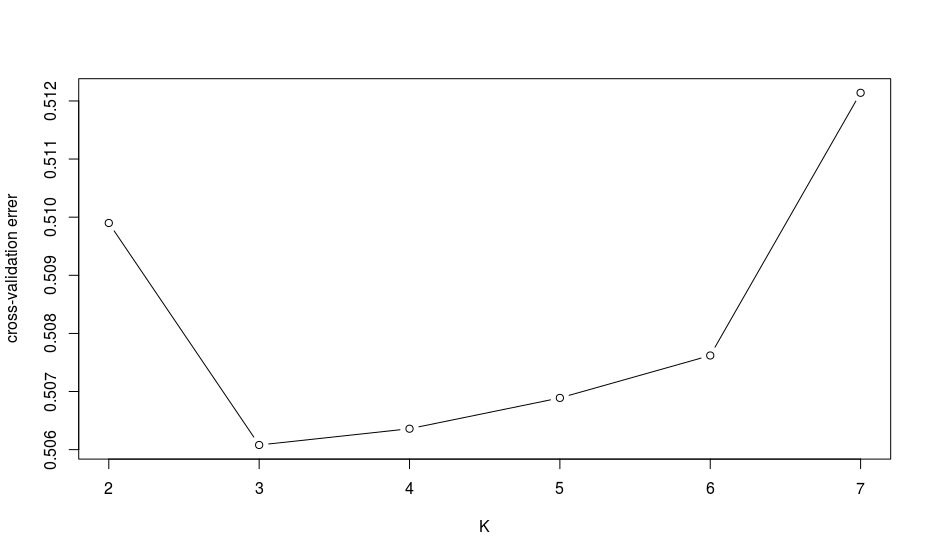

Supplement: Supplementary Figure 1 — Cross validation error in relation to the number of hypothetical ancestral populations for the Admixture analyses. [file Image_1.jpeg]

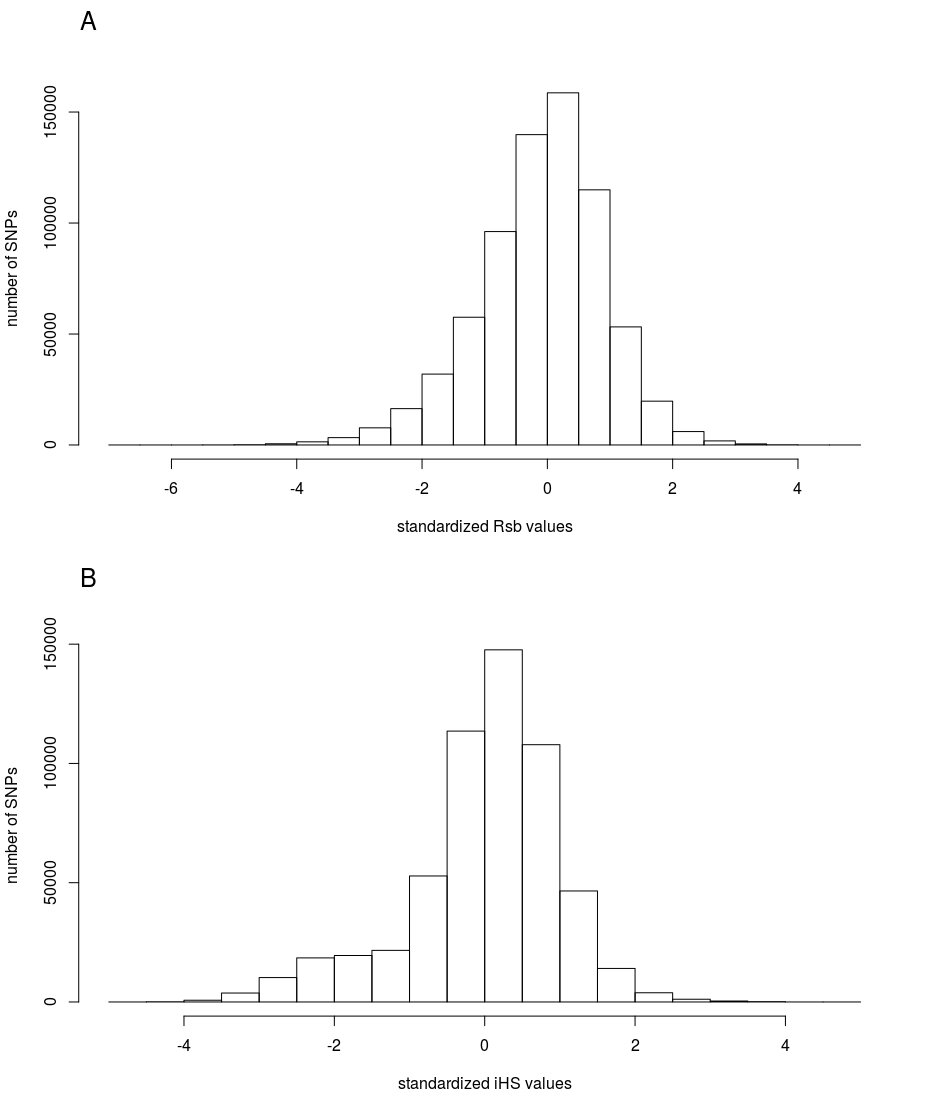

Supplement: Supplementary Figure 2 — Histogram of standardized Rsb and iHS values. [file Image_2.jpeg]

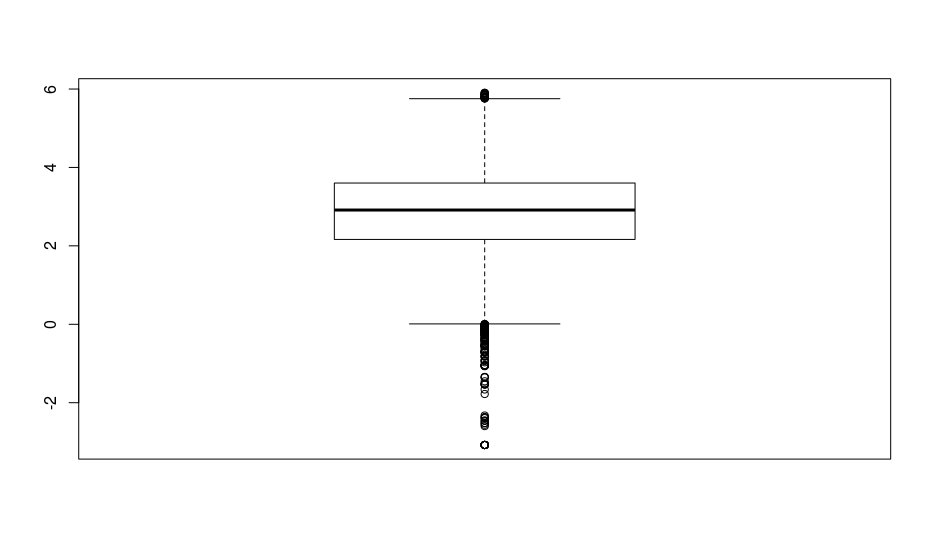

Supplement: Supplementary Figure 3 — Box plot of CLR -log (P-values). [file Image_3.jpeg]
